# Online gambling as a public health and regulatory challenge: moving beyond individual responsibility

**DOI:** 10.3389/fpubh.2026.1836481

**Published:** 2026-05-20

**Authors:** Juan S. Izquierdo-Condoy, Jorge Vasconez-Gonzalez, Esteban Ortiz-Prado

**Affiliations:** One Health Research Group, Faculty of Medicine, Universidad de las Américas, Quito, Ecuador

**Keywords:** addiction regulation, gambling disorder, health policy, online gambling, public health

## Abstract

Online gambling has expanded faster than the public health systems and regulatory frameworks designed to contain its harms. Unlike venue-based gambling, digital platforms combine constant accessibility, rapid betting cycles, frictionless payments, algorithmic personalization, and intensive marketing, features that may heighten risk and hinder early detection of problematic use. In this perspective, we argue that online gambling should be understood not only as an individual behavioral disorder, but also as a public health and regulatory challenge with broader social consequences. Recent evidence shows that online gambling is associated with gambling disorder, depression, anxiety, suicidality, financial distress, family conflict, and harms to affected others, although much of the current literature remains cross-sectional and heterogeneous. We further examine how vulnerabilities are unequally distributed across age, sex, socioeconomic position, and regional regulatory context. To move beyond a purely descriptive account, we compare recent policy responses across jurisdictions, including stake limits and a statutory levy in Great Britain, restrictive advertising rules in Belgium, and new federal controls in Brazil. These examples suggest that effective harm reduction requires more than self-regulation or individual responsibility messaging. A precautionary public health approach should include restrictions on advertising and inducements, binding deposit or loss limits, centralized exclusion systems, robust age verification, financial safeguards, and insulation of research and policymaking from industry influence.

## 1 Introduction

Gambling has evolved significantly with the advent of online platforms, which have transformed accessibility by turning mobile devices into “pocket casinos.” Since the early 2000s, digital gambling has expanded globally, removing temporal and spatial barriers inherent to traditional gambling (1). Approximately 80 million adults worldwide are estimated to experience gambling disorder, with around 448.7 million exhibiting risky gambling behaviors or suffering adverse consequences (2). The global prevalence of problem gambling among adults ranges between 0.12 and 5.8% (3). The COVID-19 pandemic intensified this trend, as lockdowns drove increased engagement in online gambling, exacerbating health-related issues and highlighting the need for stringent regulation comparable to that for addictive substances (4). The harms extend beyond the individual, affecting an average of six others in their social circle—family, friends, or colleagues—through family conflict, loss of trust, domestic violence, and mental health deterioration (5).

Online gambling has transformed the scale, speed, and visibility of gambling exposure worldwide. Mobile devices now function as always-available gambling interfaces, allowing individuals to place repeated bets across settings that are private, portable, and difficult to supervise. This matters for public health because gambling-related harm extends beyond those who meet diagnostic criteria for gambling disorder. The World Health Organization now recognizes gambling as a health issue that can contribute to mental illness, suicide, family violence, poverty, financial distress, and harms to affected others (6).

The online environment may intensify several established risk mechanisms. Compared with venue-based gambling, digital products often combine rapid event cycles, reduced friction in payment, persistent availability, and highly visible sports-linked or personalized marketing. These features may increase impulsive betting and normalize gambling as routine digital entertainment, particularly among young adults. At the same time, the relationship between gambling and mental health should be interpreted cautiously. Much of the literature remains cross-sectional, and causality is not always clear. However, newer longitudinal evidence suggests that depressive symptoms may increase the likelihood of subsequent online gambling in adolescents (7), while registry-based data also indicate elevated suicide mortality among people with gambling disorder (8).

Despite these concerns, policy responses remain uneven and frequently reactive. Public health scholars have noted that gambling regulation still tends to privilege individual responsibility over structural prevention, even as gambling becomes increasingly digital, commercialized, and integrated into everyday life (2, 9). In this perspective, we argue that online gambling should be framed as a public health and regulatory challenge shaped by commercial determinants, digital platform design, and fragmented governance. We therefore synthesize current evidence on harms, identify vulnerable populations, and compare emerging regulatory responses across jurisdictions to outline a more precautionary policy agenda.

## 2 Adverse health effects of online gambling

Online gambling has been consistently associated with a range of adverse mental health outcomes, including depression, anxiety, substance use, suicidal ideation, and broader psychosocial distress (10, 11). Comorbidities with personality disorders, obsessive-compulsive disorder (OCD), and post-traumatic stress disorder (PTSD) are also prevalent, complicating treatment and recovery (12, 13). Financial losses, shame, secrecy, and the repetitive nature of digital betting may compound these outcomes, especially when gambling becomes frequent, concealed, and difficult to interrupt (14, 15). The constant accessibility of online platforms exacerbates these issues, as individuals can gamble impulsively without external oversight, intensifying compulsive behaviors (16). Harms also extend beyond the individual gambler. Family members and partners may experience stress, relationship conflict, financial instability, and emotional burden, reinforcing the view that gambling harm is a population-level issue rather than an exclusively individual one (17). Nevertheless, because many studies are cross-sectional, these associations should not be interpreted as proof of simple one-way causation.

Physical complaints are also reported in this context, particularly sleep disturbance, fatigue, headaches, and stress-related somatic symptoms (18). These manifestations are likely to operate indirectly through chronic psychological distress, financial strain, and neglect of self-care, rather than through direct biological pathways specific to gambling itself (19). Framing these outcomes carefully helps avoid overstating the evidence while still recognizing the substantial burden associated with online gambling.

## 3 Who is most at risk—and why?

Risk is not distributed evenly across populations or settings. Young adults, particularly those aged 18–34 years and especially those between 25 and 34 years, appear to be at elevated risk of gambling-related harm in many studies, although the magnitude of this risk varies by jurisdiction, product type, and measurement approach (20). Sports betting has become especially relevant in younger populations because it is closely integrated with sport, social media, and mobile technology. Men have often been reported to experience higher rates of gambling-related problems than women, although this gap may be narrowing in some settings (21).

Socioeconomic vulnerability also plays an important role. Individuals with lower educational attainment, unemployment, unstable living conditions, or broader socioeconomic disadvantage may face a disproportionate burden of harm (22). Health inequalities can further intensify these effects: low-income individuals may encounter greater barriers to recovery, including reduced access to mental health and addiction services, while gambling-related losses may have more severe downstream consequences in financially precarious household (23). Cultural and ethnic minorities may also experience disproportionate harm because of targeted marketing and unequal access to support services (24).

Importantly, vulnerability is not only demographic but also structural. Online gambling products differ from one another, and their design features—continuous accessibility, in-play betting, repeated prompts, personalization, and reduced payment friction—may interact with pre-existing mental health symptoms, social stress, and financial precarity. Exposure to gambling advertising is also a key contextual factor, as recent systematic evidence suggests that gambling promotion may shape attitudes, normalize participation, and increase gambling behavior, particularly among already vulnerable groups (25). This is why a purely “responsible gambling” framing is insufficient. The question is not only who gambles, but under what digital, commercial, and regulatory conditions gambling becomes more harmful.

## 4 Public health implications

The main public health concern is not simply that online gambling is available, but that it is increasingly embedded in digital ecosystems designed for continuous engagement. Its distinctive features—constant accessibility, relative anonymity, rapid betting cycles, reduced payment friction, and limited human supervision—may amplify harmful use compared with many venue-based forms of gambling (26). In digital environments, problematic gambling can develop with less social visibility and fewer opportunities for early interruption. The use of electronic or cashless payment systems may further reduce awareness of financial losses, while the absence of direct oversight can hinder early recognition of escalating risk. Younger individuals, particularly adolescents and young adults, may be especially vulnerable in these settings because of their greater exposure to online platforms, sports-linked betting cultures, and digital advertising (26, 27).

The resulting harms extend well beyond clinical disorder. They include debt, impaired work and academic performance, family disruption, and increased demand for mental health, addiction, and social support services. In more severe cases, gambling-related harm may contribute to personal bankruptcy, domestic conflict, and other downstream social consequences driven by financial desperation and psychosocial deterioration (28). The burden is therefore distributed across households, communities, and health systems, not solely among individuals who meet formal diagnostic thresholds (17).

This broader framing matters because it shifts the policy question from treatment alone to prevention, regulation, surveillance, and commercial accountability (2, 6, 29). Recent public health scholarship argues that gambling should be approached similarly to other harmful commercial products: through upstream regulation, not only downstream counseling. This includes attention to advertising, platform design, inducements, payment systems, age verification, and the influence of industry on research and policymaking (2, 6, 29).

## 5 Regulations and call to action

Regulatory responses to online gambling remain fragmented, but recent developments across jurisdictions provide useful lessons. In Great Britain, the government has moved toward stronger structural protections by introducing a statutory levy on gambling operators and online slot stake limits, including lower limits for young adults (30). In Belgium, the Royal Decree of 27 February 2023 established very strict rules on gambling advertising (31); however, emerging post-ban data suggest that substantial exposure to gambling advertising may persist, highlighting the importance of enforcement and the challenge of digital spillover (32). In Brazil, the new federal betting framework taking effect in 2025 introduced operator authorization, restrictions on betting with credit, tighter identification requirements, and stronger protections against underage participation (33). By contrast, the United States continues to illustrate the risks of fragmented expansion, where rapid growth in sportsbook access has outpaced consistent public health safeguards (34).

These examples suggest that isolated measures are unlikely to be sufficient (29). Effective policy should combine restrictions on advertising and inducements, binding deposit or loss limits, centralized exclusion systems, meaningful age and identity verification, financial transaction safeguards, independent monitoring, and stable funding for prevention, treatment, and research through taxation or statutory levies (2, 6, 29). A precautionary model is especially important where markets are expanding faster than local evidence systems or treatment capacity (2, 29). The goal should not be to rely primarily on individual self-control within highly engineered digital environments, but to redesign those environments so that harm becomes less likely at the population level (2, 6, 9).

## 6 Limitations of this perspective

This manuscript has several limitations. Much of the evidence base on which it relies is still derived from cross-sectional studies, which constrains causal inference and makes bidirectional relationships plausible. In addition, online gambling is a heterogeneous phenomenon, and the associated risks may differ substantially across sports betting, online casinos, poker, and other digitalized forms of gambling, making accurate measurement and comparison more challenging. Third, regulatory environments are evolving rapidly, meaning that policy comparisons may change over time. Finally, literature remains disproportionately concentrated in high-income countries, whereas evidence from other settings, including Latin America and other low- and middle-income contexts, remains comparatively limited despite rapid market growth. This gap hinders both the characterization of the problem and evidence-based decision-making. Taken together, these limitations underscore the need for more longitudinal research, as well as studies focused on developing regions that can better capture the realities and effects of online gambling in these populations. They also highlight the importance of jurisdiction-specific research and policy evaluation studies addressing online gambling regulation.

## 7 Conclusions

Online gambling poses significant risks to individual and public health, driven by its accessibility, anonymity, and addictive design. It is associated with severe mental and physical health issues, including anxiety, depression, substance use disorders, and physical ailments linked to stress. Vulnerable groups, such as young adults, men, and low-income individuals, face heightened risks, with broader societal impacts including family distress, economic losses, and increased demand on healthcare systems. Strict, coordinated regulations are essential to limit industry influence, protect at-risk populations, and mitigate the profound health and social consequences of online gambling.

## Data Availability

The original contributions presented in the study are included in the article/supplementary material, further inquiries can be directed to the corresponding author.

## References

[1] Gainsbury SM. Online gambling addiction: the relationship between internet gambling and disordered gambling. Curr Addict Rep. (2015) 2:185–93. doi: 10.1007/s40429-015-0057-8 26500834 PMC4610999

[2] Wardle H Degenhardt L Marionneau V Reith G Livingstone C Sparrow M . The lancet public health commission on gambling. Lancet Public Health. (2024) 9:e950–94. doi: 10.1016/S2468-2667(24)00167-1 39491880

[3] Calado F Griffiths MD. Problem gambling worldwide: an update and systematic review of empirical research (2000–2015). J Behav Addict. (2016) 5:592–613. doi: 10.1556/2006.5.2016.073 27784180 PMC5370365

[4] Håkansson A Fernández-Aranda F Menchón JM Potenza MN Jiménez-Murcia S. Gambling during the COVID-19 crisis—a cause for concern. J Addict Med. (2020) 14:e10–2. doi: 10.1097/ADM.0000000000000690 32433365 PMC7273946

[5] Goodwin BC Browne M Rockloff M Rose J. A typical problem gambler affects six others. Int Gambling Stud. (2017) 17:276–89. doi: 10.1080/14459795.2017.1331252

[6] World Health Organization. Gambling. Geneva: World Health Organization (2024). Available online at: https://www.who.int/news-room/fact-sheets/detail/gambling (Accessed September 10, 2025).

[7] Pilkington ML Gohari MR Cole AG Ferro MA Elton-Marshall T Laxer R . Prospective study of mental health in relation to online gambling one-year later in a large cohort of adolescents in Canada. J Gambl Stud. (2026) 42:235–47. doi: 10.1007/s10899-025-10447-2 41171599 PMC13009133

[8] Kristensen JH Baravelli CM Leino T Pallesen S Griffiths MD Erevik EK. Association between gambling disorder and suicide mortality: a comparative cohort study using Norwegian health registry data. Lancet Reg Health Eur. (2025) 48. doi: 10.1016/j.lanepe.2024.0101127 PMC11600009 39606747

[9] Ukhova D Marionneau V Nikkinen J Wardle H. Public health approaches to gambling: a global review of legislative trends. Lancet Public Health. (2024) 9:e57–67. doi: 10.1016/S2468-2667(23)00221-9 37944544 PMC10927617

[10] Moghaddam JF Yoon G Dickerson DL Kim SW Westermeyer J. Suicidal ideation and suicide attempts in five groups with different severities of gambling: findings from the National Epidemiologic Survey on alcohol and related conditions. Am J Addict. (2015) 24:292–8. doi: 10.1111/ajad.12197 25808267

[11] Dowling NA Jackson AC Suomi A Lavis T Thomas SA Patford J . Problem gambling and family violence: prevalence and patterns in treatment-seekers. Addict Behav. (2014) 39:1713–7. doi: 10.1016/j.addbeh.2014.07.006 25117847

[12] Hing N Russell AM Browne M. Risk factors for gambling problems on online electronic gaming machines, race betting and sports betting. Front Psychol. (2017) 8:779. doi: 10.3389/fpsyg.2017.00779 28555121 PMC5430067

[13] Parhami I Siani A Rosenthal RJ Fong TW. Pathological gambling, problem gambling and sleep complaints: an analysis of the National Comorbidity Survey: Replication (NCS-R). J Gambl Stud. (2013) 29:241–53. doi: 10.1007/s10899-012-9299-8 22396174 PMC3395730

[14] Welte JW Barnes GM Tidwell M-CO Hoffman JH. Gambling and problem gambling across the lifespan. J Gambl Stud. (2011) 27:49–61. doi: 10.1007/s10899-010-9195-z 20499144 PMC4383132

[15] Afifi TO Cox BJ Martens PJ Sareen J Enns MW. The relationship between problem gambling and mental and physical health correlates among a nationally representative sample of Canadian women. Can J Public Health. (2010) 101:171–5. doi: 10.1007/BF03404366 20524385 PMC6973819

[16] Suomi A Jackson AC Dowling NA Lavis T Patford J Thomas SA . Problem gambling and family violence: family member reports of prevalence, family impacts and family coping. Asian J. Gambl. Issues Public Health. (2013) 3:13. doi: 10.1186/2195-3007-3-13

[17] Thorley C Stirling A Huynh E. Cards on the Table: the Cost to Government Associated With People Who are Problem Gamblers in Britain. London: Institute for Public Policy Research (2016). Available online at: https://www.ippr.org/articles/cards-on-the-table (Accessed September 20, 2025).

[18] Hing N Breen H Gordon A Russell A. Gambling harms and gambling help-seeking amongst indigenous Australians. J Gambl Stud. (2014) 30:737–55. doi: 10.1007/s10899-013-9388-3 23740348

[19] King DL Delfabbro PH. Adolescents' perceptions of parental influences on commercial and simulated gambling activities. Int Gambl Stud. (2016) 16:424–41. doi: 10.1080/14459795.2016.1220611

[20] Browne M Greer N Armstrong T Doran C Kinchin I Langham E . The Social Cost of Gambling to Victoria. Melbourne: Victorian Responsible Gambling Foundation (2017). Available online at: https://responsiblegambling.vic.gov.au/resources/publications/the-social-cost-of-gambling-to-victoria-121/ (Accessed September 20, 2025).

[21] Gainsbury SM Russell A Hing N Wood R Lubman DI Blaszczynski A. The prevalence and determinants of problem gambling in Australia: assessing the impact of interactive gambling and new technologies. Psychol Addict Behav. (2014) 28:769–79. doi: 10.1037/a0036207 24865462

[22] Productivity Commission Australian Government. Gambling: Productivity Commission Inquiry Report. Australian Government (2010). Available online at: https://www.pc.gov.au/inquiries/completed/gambling-2010/report (Accessed September 20, 2025).

[23] Lorains FK Cowlishaw S Thomas SA. Prevalence of comorbid disorders in problem and pathological gambling: systematic review and meta-analysis of population surveys. Addiction. (2011) 106:490–8. doi: 10.1111/j.1360-0443.2010.03300.x 21210880

[24] Livingstone C Adams PJ. Clear principles are needed for integrity in gambling research. Addiction. (2016) 111:5–10. doi: 10.1111/add.12913 26058407

[25] McGrane E Pryce R Field M Gu S Moore EC Goyder E. What is the impact of sports-related gambling advertising on gambling behaviour? A systematic review. Addiction. (2025) 120:589–607. doi: 10.1111/add.16761 39797658 PMC11907332

[26] Hing N Browne M Russell AMT Rockloff M Rawat V Nicoll F . Avoiding gambling harm: an evidence-based set of safe gambling practices for consumers. PLoS ONE. (2019) 14:e0224083. doi: 10.1371/journal.pone.0224083 31622430 PMC6797237

[27] Auer MM Griffiths MD. The use of personalized behavioral feedback for online gamblers: an empirical study. Front Psychol. (2015) 6:1406. doi: 10.3389/fpsyg.2015.01406 26441779 PMC4585278

[28] Adams PJ Rossen F. A tale of missed opportunities: pursuit of a public health approach to gambling in New Zealand. Addiction. (2012) 107:1051–6. doi: 10.1111/j.1360-0443.2012.03800.x 22563834

[29] Fisher ML Piper T Fitzpatrick M Mavi S Retzer A Bradbury-Jones C . Legal and regulatory responses to online gambling harms: a scoping review of evidence. Harm Reduct J. (2025) 22:163. doi: 10.1186/s12954-025-01292-y 41063227 PMC12505849

[30] Department for Culture Media and Sport and Baroness Twycross UK Government. Statutory Levy and Online Slot Stake Limits to be Introduced to Tackle Gambling Harm. GOVUK (2024). Available online at: https://www.gov.uk/government/news/statutory-levy-and-online-slot-stake-limits-to-be-introduced-to-tackle-gambling-harm (Accessed April 22, 2026).

[31] Belgian Gaming Commission. Article 61—Royal Decree of 27 February 2023 Determining the Conditions for Advertising Games of Chance. FPS Gaming Commission (2023). Available online at: https://www.ejustice.just.fgov.be/mopdf/2023/03/08_3.pdf#Page2 (Accessed April 22, 2026).

[32] De Jans S Hudders L. Newall P. Gambling advertising still exists in Belgium despite a widely reported “ban”. Addiction. (2024) 119:1324–6. doi: 10.1111/add.16458 38433492

[33] Secretaria de Comunicação Social da Presidência da República. Regulations introduced by the Secretariat of Prizes and Betting will place Brazil in a regulated betting market by 2025. gov.br (2024). Available online at: https://www.gov.br/secom/en/latest-news/2024/12/regulations-introduced-by-the-secretariat-of-prizes-and-betting-will-place-brazil-in-a-regulated-betting-market-by-2025 (Accessed April 22, 2026).

[34] Yeola A Allen MR Desai N Poliak A Yang KH Smith DM . Growing health concern regarding gambling addiction in the age of sportsbooks. JAMA Intern Med. (2025) 185:382–9. doi: 10.1001/jamainternmed.2024.8193 39960737 PMC11833657

